# Supplementary material for: TREM2 shedding by cleavage at the H157‐S158 bond is accelerated for the Alzheimer's disease‐associated H157Y variant
Source: EMBO Mol Med. 2017 Aug 30;9(10):1366–78. doi: 10.15252/emmm.201707673 (PMC5623839; doi:10.15252/emmm.201707673)
Supplement: Supplementary file 1 — Expanded View Figures PDF [file EMMM-9-1366-s001.pdf]

Expanded View Figures

Supernatant

Time (h)	biotin TREM2 [ng]					Time (h)	% t=0	SEM
0.0	0.0	0.0	0.0	0.0		0.0	0.0	0.0
0.5	188.7	314.4	881.6	651.2		0.5	112.2	34.8
1.0	267.8	407.4	767.7	972.8		1.0	133.1	35.7
4.5	182.6	316.3	502.7	644.0		4.5	90.7	22.4

Membrane

Time (h)	biotin TREM2 [ng]					Time (h)	% t=0	SEM
0.0	346.3	338.2	545.2	585.3		0.0	100.0	14.3
0.5	115.9	115.5	244.4	254.5		0.5	40.2	8.5
1.0	127.9	130.1	134.3	188.2		1.0	32.0	3.2
4.5	131.4	114.5	95.4	129.5		4.5	25.9	1.8

Cytosol

Time (h)	biotin TREM2 [ng]					Time (h)	% t=0	SEM
0.0	116.1	150.0	431.2	417.7		0.0	61.4	18.6
0.5	53.5	64.9	141.0	159.3		0.5	23.1	5.9
1.0	32.8	39.1	102.5	112.6		1.0	15.8	4.6
4.5	28.3	26.3	23.6	26.0		4.5	5.7	0.2

Nucleus

Time (h)	biotin TREM2 [ng]					Time (h)	% t=0	SEM
0.0	128.1	122.5	289.3	323.6		0.0	47.6	11.6
0.5	119.1	114.8	270.3	287.8		0.5	43.6	10.3
1.0	144.8	99.9	196.5	250.5		1.0	38.1	7.2
4.5	119.1	84.5	161.0	174.8		4.5	29.7	4.5

Figure EV1. Surface biotinylation and fate of TREM2.

Surface-expressed TREM2 on human macrophages was biotinylated at $t = 0$ and fractionated into four pools: supernatant, membrane-associated, cytosolic and nuclear. Similar fractionation was undertaken following incubation for 0.5, 1.0 and 4.5 h. TREM2-associated biotin was purified by immunoprecipitation and quantified by MSD for biotinylated TREM2. The raw and processed data are presented here. The left block of data shows biotin TREM2 measurements for four timepoints for four biological replicates for each of the four subcellular pools. The right block of data represents the normalised TREM2 quantification, with the membrane fraction at $t = 0$ defined as 100%.

 © 2017 MedImmune Ltd

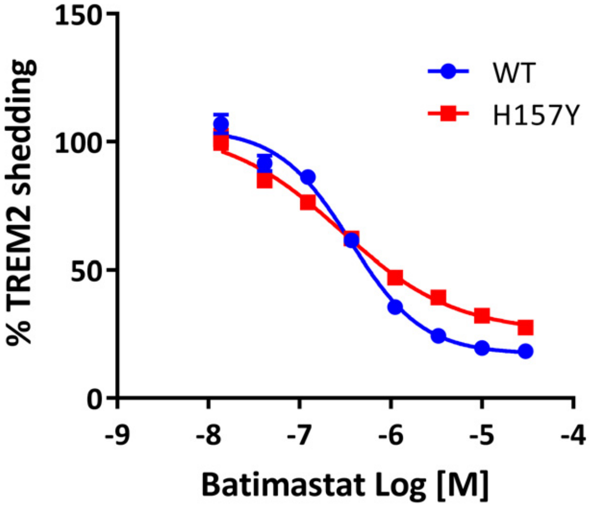

Figure EV2. Titration of batimastat against WT and H157Y TREM2 shedding.

The concentration of batimastat in the culture medium of HEK293 cells expressing either WT or H157Y TREM2 (with hDAP12) was varied over 4.5 orders of magnitude and shed TREM2 quantified bt MSD assay 24 h later. More batimastat-resistant shedding of the variant TREM2 is apparent at higher inhibitor concentrations as compared to the WT protein. Data plotted as mean ± SEM.

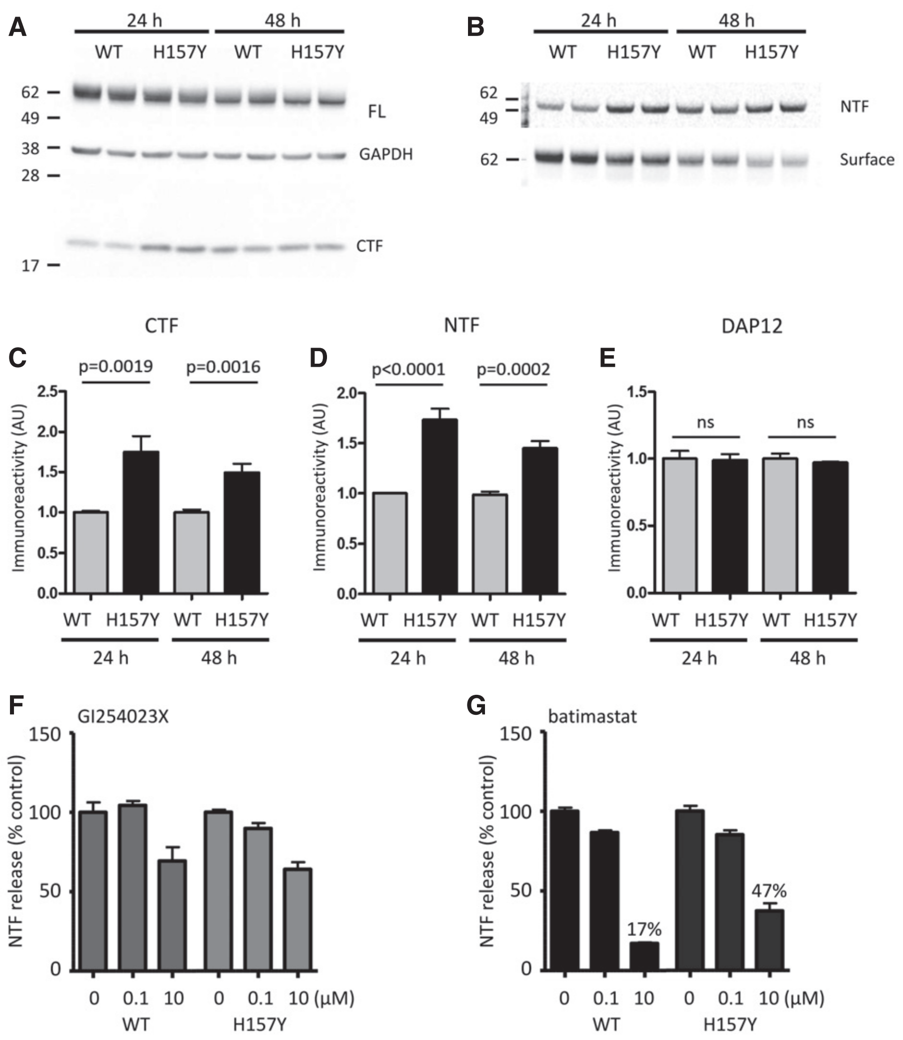

Figure EV3. The disease-linked H157Y variant of TREM2 is shed more rapidly from the cell surface.

A–E (A) Western blot of HEK293-HaloTag-hTREM2 cells demonstrating full-length protein (FL) and also levels of the TREM2 C-terminal fragment (CTF) that were higher for the H157Y variant (H157Y) as compared to wild type, particularly at 24 h (quantified in C). (B) Likewise, the NTF from the same cells accumulated more in the H157Y variant-expressing cell supernatants (quantified in D). (E) Levels of DAP12, expressed from the same plasmid, were unchanged.

F, G Inhibition of shedding by GI254023X was equivalent for both isoforms of TREM2 (F); however, the metalloprotease inhibitor batimastat (G) was less effective at blocking shedding of the H157Y variant.

Data information: Data plotted as mean ± SEM. Two-tailed Student's *t*-test.
Source data are available online for this figure.

© 2017 MedImmune Ltd

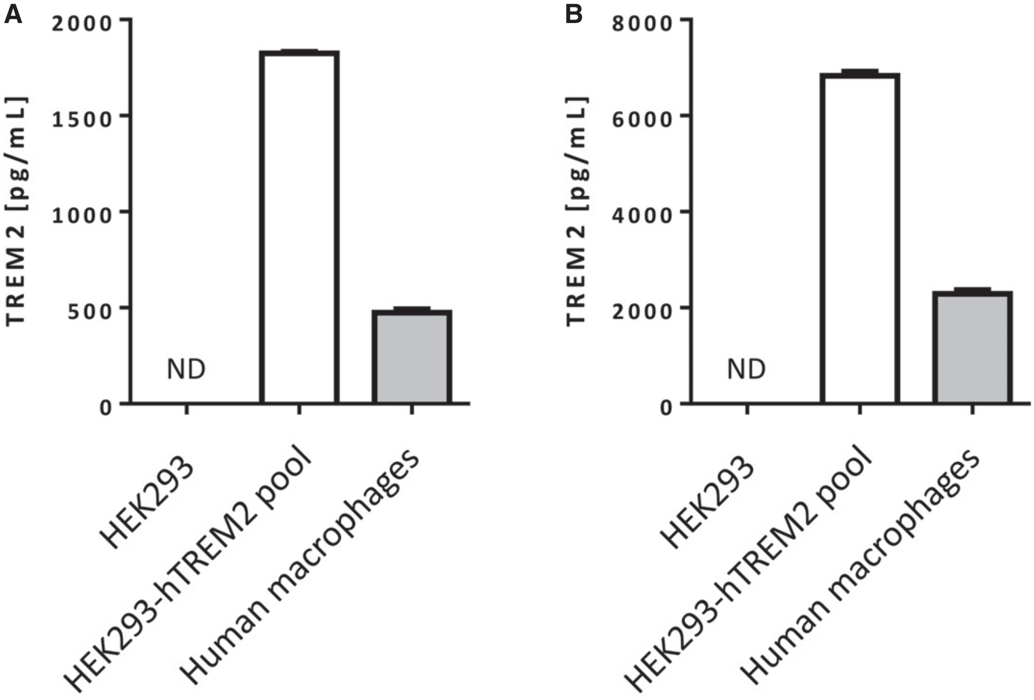

Figure EV4. Validation data for the shed TREM2 MSD assay.

A, B The MSD assay detected TREM2 in the conditioned media (A) and lysates (B) of HEK293 cells stably expressing hTREM2 and of primary human macrophages but not in parental HEK293 cultures. ND = not detected.

 © 2017 MedImmune Ltd
