# Supplementary figures and images for: TREM2 shedding by cleavage at the H157‐S158 bond is accelerated for the Alzheimer's disease‐associated H157Y variant
Source: EMBO Mol Med. 2017 Aug 30;9(10):1366–78. doi: 10.15252/emmm.201707673 (PMC5623839; doi:10.15252/emmm.201707673)

FigEV3A: chemiluminescence green  
channel

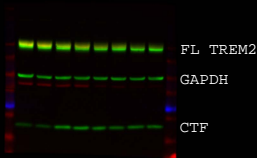

FigEV3B

TREM2 NTF

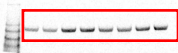

# FigEV3B TREM2 surface

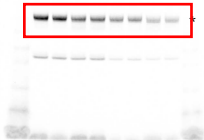

Supplement: Supplementary file 2 — Source Data for Expanded View [file EMMM-9-1366-s007.zip › EMM_07673_Source_data_FigEV3.pdf]

Figure 1A

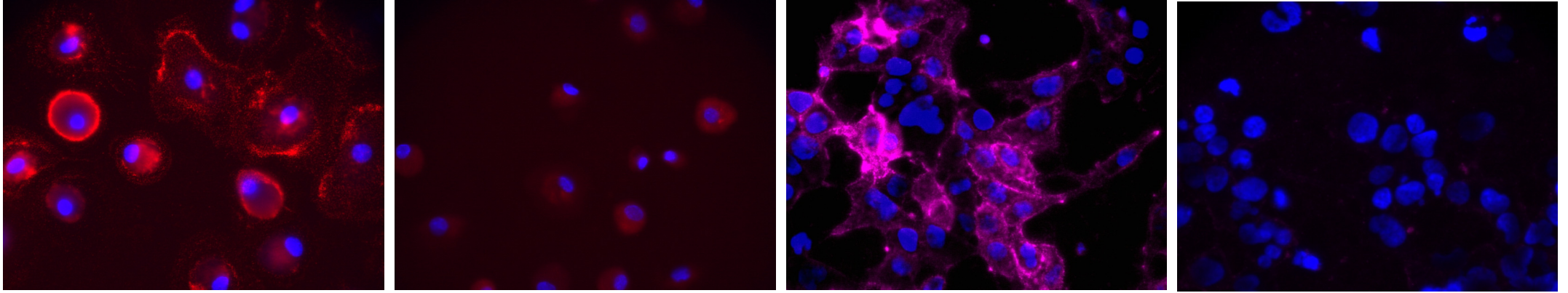

Figure 1B

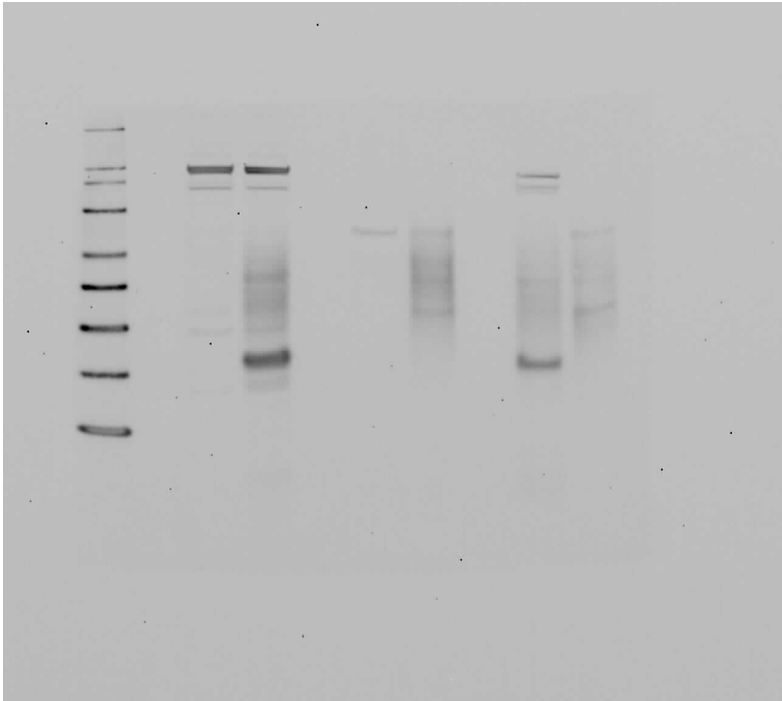

Supplement: Supplementary file 4 — Source Data for Figure 1 [file EMMM-9-1366-s002.pdf]

Figure 2A

Part of image used

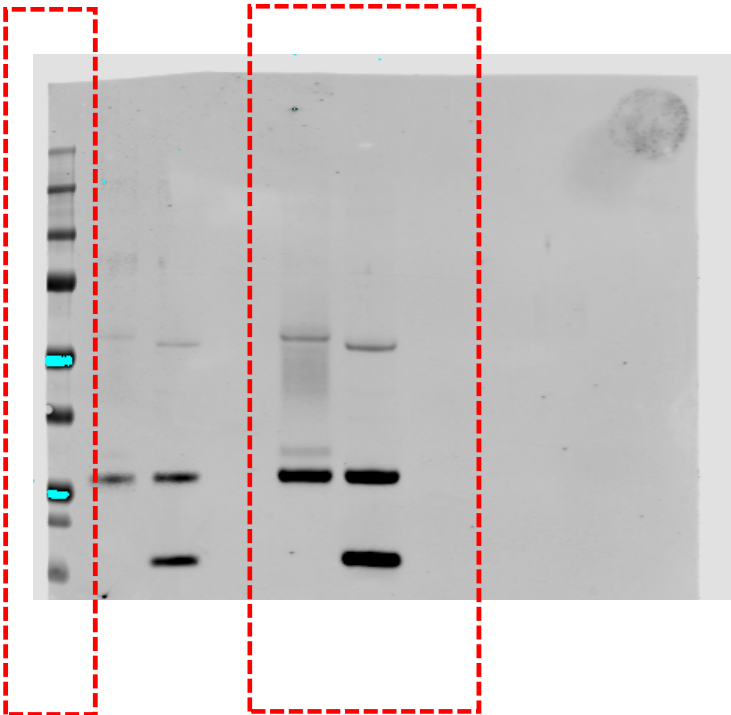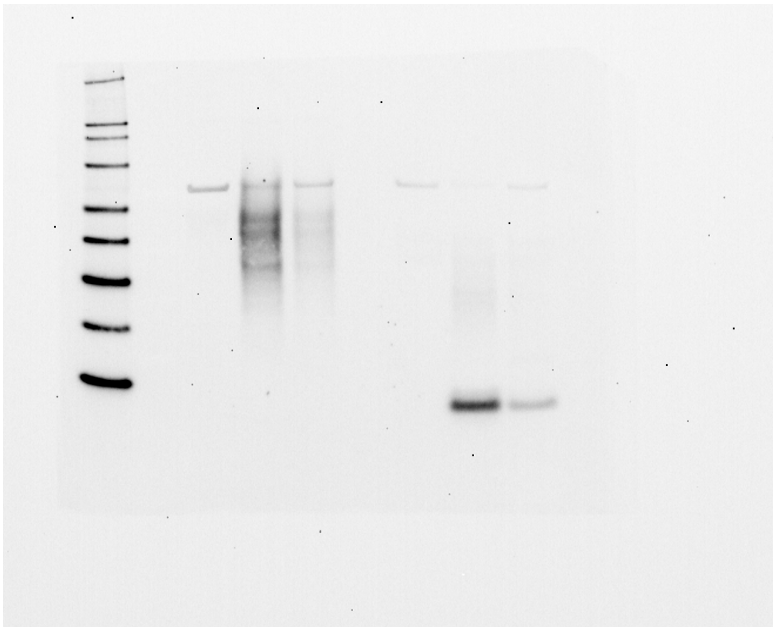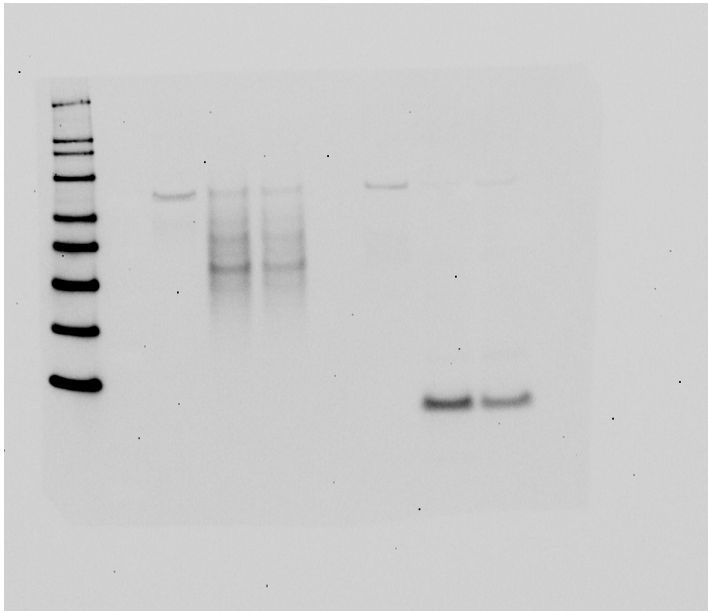

Supplement: Supplementary file 5 — Source Data for Figure 2 [file EMMM-9-1366-s003.pdf]

Figure 4A

Part of image used

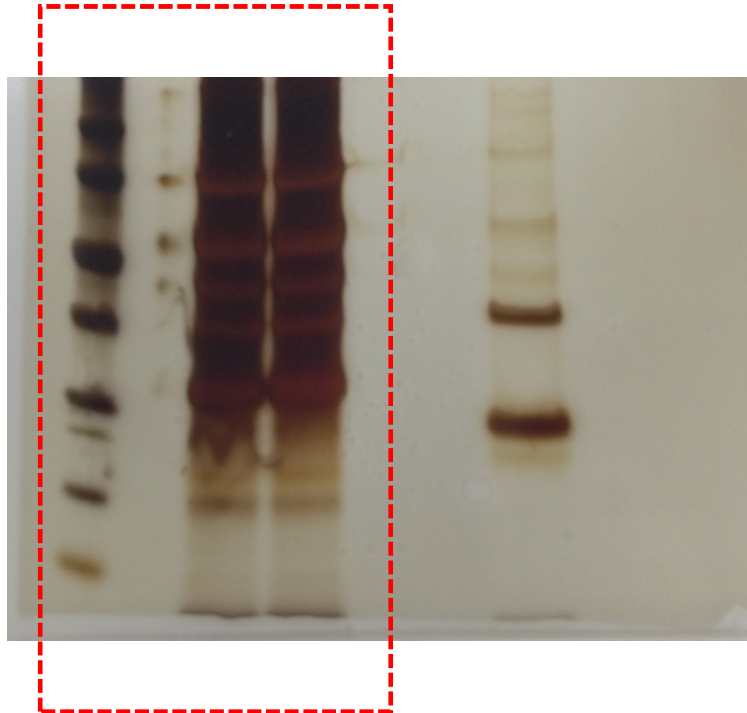

Supplement: Supplementary file 6 — Source Data for Figure 4 [file EMMM-9-1366-s004.pdf]

Fig5A

DAP12

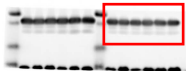

Fig5A

TREM2

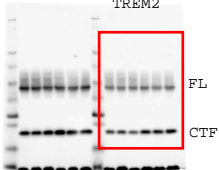

Fig5A

GAPDH

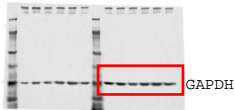

Fig5C

sAPPalpha

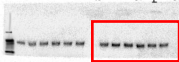

sAPPa

Fig5C

TREM2 NTF

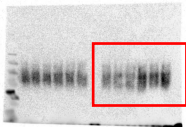

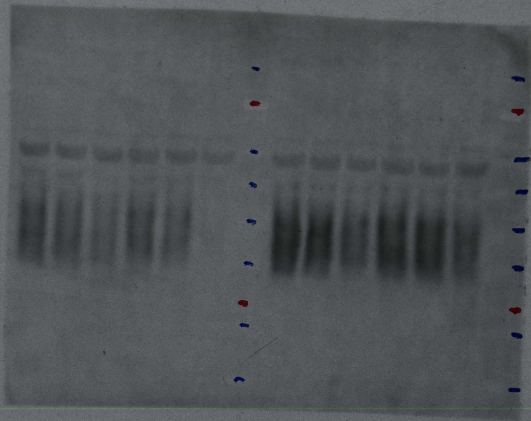

2017.01.31

$\alpha$  NTF T<sub>2</sub>

60 d

TREM2 Fig5E

Supplement: Supplementary file 7 — Source Data for Figure 5 [file EMMM-9-1366-s005.pdf]

Fig6A

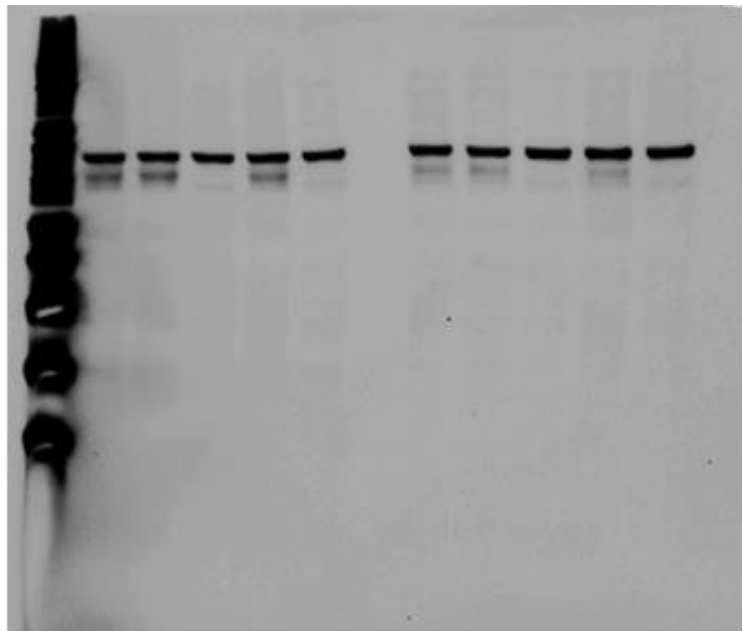

Fig6B

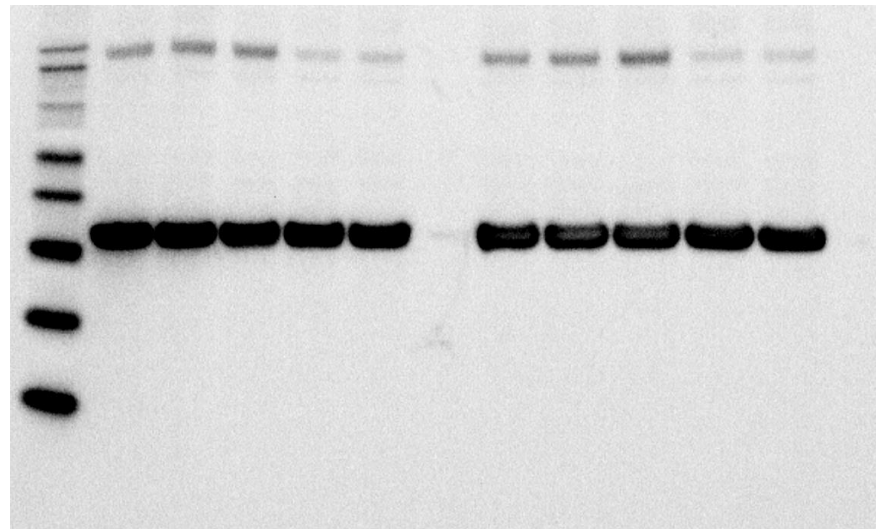

Supplement: Supplementary file 8 — Source Data for Figure 6 [file EMMM-9-1366-s006.pdf]
